# Application and development of Deuterium Metabolic Imaging in tumor glucose metabolism: visualization of different metabolic pathways

**DOI:** 10.3389/fonc.2023.1285209

**Published:** 2023-11-28

**Authors:** Jiayu Wan, Yusheng Guo, Hebing Chen, Peng Sun, Xiaoxiao Zhang, Tianhe Ye, Lingli Li, Feng Pan, Lian Yang

**Affiliations:** ^1^ Department of Radiology, Union Hospital, Tongji Medical College, Huazhong University of Science and Technology, Wuhan, China; ^2^ Hubei Key Laboratory of Molecular Imaging, Wuhan, China; ^3^ Clinical Technical Solutions, Philips Healthcare, Beijing, China

**Keywords:** cancer metabolism, magnetic resonance imaging, molecular imaging, deuterium metabolic imaging (DMI), cancer immunotherapy

## Abstract

Cancer metabolism has emerged as a pivotal area of research recently. The ability to visualize and comprehend the metabolic processes of cancer holds immense clinical value, particularly in the diagnosis of malignant tumors and the assessment of treatment responses. Deuterium Metabolic Imaging (DMI), as a robust, simple, and versatile MR spectroscopic imaging tool, demonstrates promise in tumor diagnosis and treatment efficacy assessment. This review explored the latest developments and applications of DMI in oncology across various tumor metabolic axes, with a specific emphasis on its potential for clinical translation. DMI offers invaluable insights into tumor biology, treatment responses, and prognostic outcomes. Notably, DMI can identify early responses to immunotherapy, a prominent area of current research interest. In conclusion, DMI harbors the potential to evolve into a convenient and efficient imaging technique in clinical practice, thereby advancing precision medicine and improving the diagnosis and evaluation of cancer treatments.

## 1 Introduction

Metabolism has long been a topic of interest in cancer research, due to the distinct metabolic traits that tumor cells exhibit compared to their normal counterpart. These metabolic adaptations, encompassing various intracellular and extracellular alterations, play a pivotal role not only in the growth and proliferation of malignant cells but also in shaping gene expression, cellular differentiation, and the tumor microenvironment (1–4). Among these alterations, glucose metabolic reprogramming holds particular significance in tumor cells, leading to increased glucose uptake and consumption (5). This metabolic reprogramming opens up new avenues for malignancy treatment, including the use of glycolytic inhibitors such as 2-deoxy-D-glucose and 3-bromopyruvate (6–8). Consequently, understanding and visualizing aberrant tumor metabolism offers immense potential for non-invasive malignancy diagnosis and treatment assessment.

The advancement of precision medicine in modern oncology has driven the development of biomedical imaging techniques and functional metabolic therapies, enabling comprehensive investigations of tumor biology at the cellular and molecular levels (9–11). Recently, there has been an increasing emphasis on imaging tumor tissue metabolism, leading to dedicated studies in this field (4, 12). Among the various metabolic imaging techniques, such as Positron Emission Computed Tomography (PET), dynamic hyperpolarized ^13^C Magnetic Resonance Spectroscopy Imaging (MRSI), and Deuterium Metabolic Imaging (DMI), DMI stands out as a versatile and robust approach that provides more flexible dosing and scanning time by using ^2^H-enriched metabolites with high homology to endogenous molecules (13). Its simplicity, including the use of MR (magnetic resonance) methods, obviates the need for water and lipid signal suppression, making it inherently robust for metabolic imaging (14). Furthermore, the low gyromagnetic ratio of the ^2^H isotope coupled with the sparse MR spectrum of ^2^H-containing metabolic molecules renders DMI minimally affected by magnetic field inhomogeneity (14). Additionally, favorable T1 and T2 relaxation times of DMI further facilitate high sensitivity through fast scanning with excellent spectral resolution (15). The availability of a wide range of non-radioactive and biocompatible ^2^H-enriched substrates targeting various metabolic pathways has further propelled the development of DMI, making it a promising tool in oncology. Hence, this review focuses on recent advancements and applications of DMI in oncology, highlighting its potential for clinical translation, and discussing its future development trends.

## 2 Existing imaging technologies and comparison

Currently, clinical diagnosis and treatment monitoring of cancer heavily rely on PET imaging, primarily using 2-^18^F-fluoro-2-deoxy-D-glucose (^18^FDG) as the imaging agent. PET exploits the increased glycolysis in tumor tissue, allowing for the detection of tumor uptake and phosphorylation of FDG (16–19). It plays a crucial role in tumor diagnosis, staging, and assessing treatment response, while also aiding in identifying and monitoring immune-related toxicities (17, 19). However, PET has several limitations: First, PET provides high-resolution maps of glucose uptake but is limited in detecting downstream metabolites; additionally, it may encounter challenges in identifying tumors in organs with high baseline glucose uptake, such as the brain, resulting in decreased contrast in brain tumor imaging (20, 21). Second, ^18^FDG is a radioactive tracer that limits longitudinal measurements and may prohibit examination in certain patient populations due to safety concerns.

Hyperpolarized ^13^C MRSI is an emerging magnetic resonance spectroscopy imaging technique that significantly enhances the magnetic resonance signal by utilizing dynamic nuclear polarization (DNP) (22). This technology holds promise for early-stage cancer diagnosis and treatment response monitoring, particularly in prostate cancer, breast cancer, renal clear cell tumors, and central nervous system tumors (23–27). The primary advantage of hyperpolarized ^13^C MRSI is its ability to detect downstream metabolites of glucose. However, its clinical implementation faces challenges due to additional hardware requirements, lengthy operating time and associated high costs (28).

**Figure 1:**
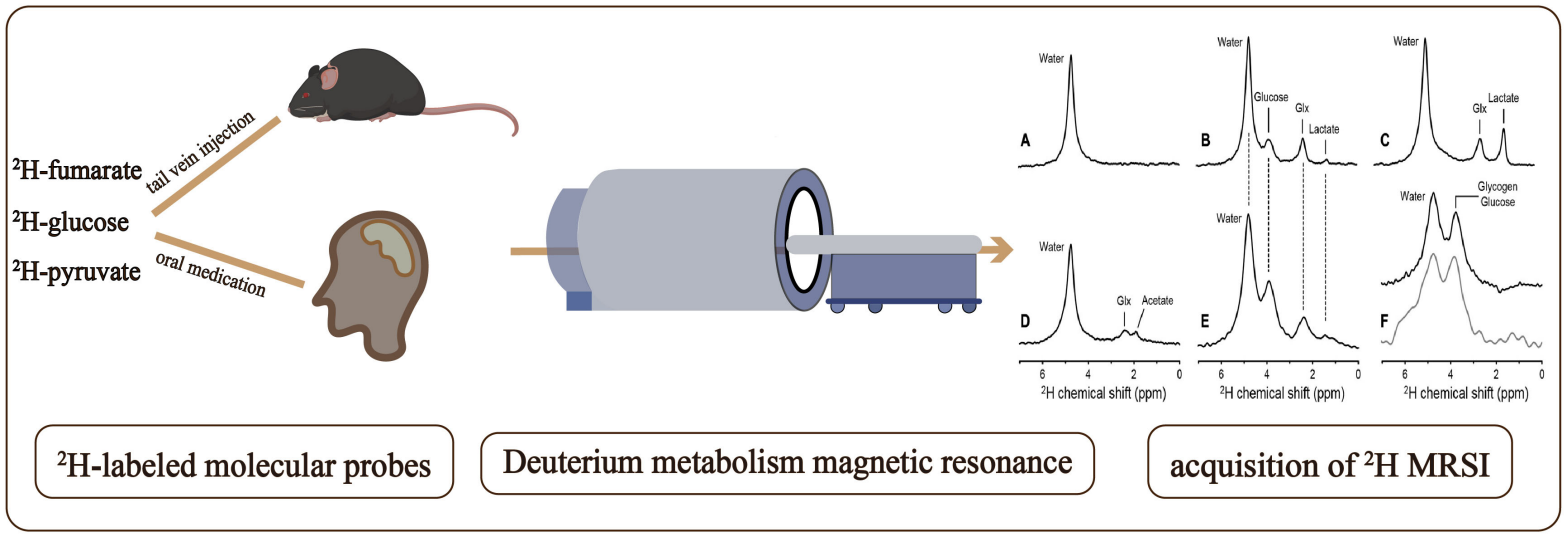
Workflow of a Deuterium Metabolic Imaging (DMI) study: Following the administration of deuterium-labeled glucose, via tail vein injection in mice and oral ingestion in humans, deuterium metabolism magnetic resonance imaging (DMSI) is employed for signal detection. The obtained data is subsequently subjected to comprehensive processing and analysis as shown in **(A–F)** to facilitate interpretation. Deuterium NMR spectra acquired without localization from **(A)** rat brain in vivo at 11.7 T before infusion of any 2H-labeled substrate, **(B)** rat brain after infusion of 2H-glucose in vivo, **(C)** rat brain after infusion of 2H-glucose postmortem, and **(D)** rat brain after infusion of 2H-acetate in vivo. **(E)** 2H NMR spectrum acquired from human brain at 4 T, 60 min after oral administration of 2H-glucose. **(F)** 2H NMR spectra from rat (top, black) and human (bottom, gray) liver after intravenous and oral administration of 2H-labeled glucose, respectively. **(A-F)** are cited from De Feyter et al. (29) (non-commercial 4.0 international license, CC BY-NC 4.0).

DMI employs magnetic resonance to detect ^2^H-labeled molecular probes, and related metabolites as illustrated in **Figure 1**. By analyzing the downstream metabolites of these probes, DMI can visualize various metabolic pathways and provide molecular insights into tumor tissue metabolism without radiation exposure (30). Incorporating spatial localization coding allows for the analysis and comparison of the concentration of related ^2^H-labeled metabolites in different tissues (30). Due to the lower chemical shift range, J-coupling constants, and coupling effects of deuterium compared to hydrogen, the complexity of deuterium spectrum is simplified, which facilitates the interpretation and analysis of DMI and enables researchers to focus on specific deuterium labeled metabolites and their metabolic kinetics (14, 29). DMI enables dynamic tracking of the entire process of cell metabolism within tumor cells, including glucose transport, pentose 6-phosphate pathway conversion (PPP), glycolysis, and the Krebs cycle (31). These capabilities hold significant potential for guiding precision treatments (29).

**Figure 2:**
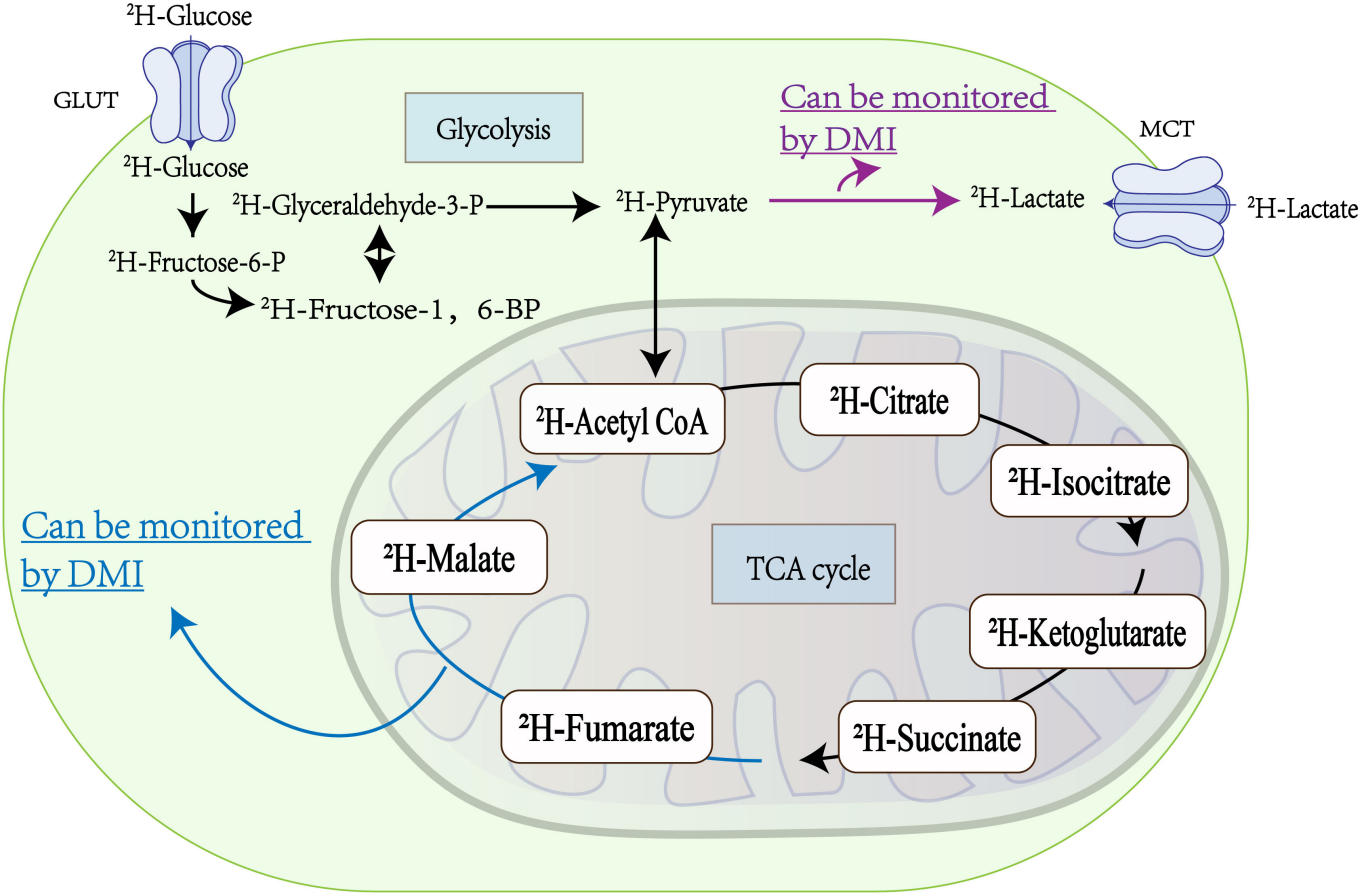
Metabolic processes within tumor cells: Glucose ingress into tumor cells via the glucose transporter (GLUT) initiates two vital metabolic pathways, glycolysis and the tricarboxylic acid (TCA) cycle. Within tumor cells, glycolysis predominates as the primary energy source, resulting in the substantial production of lactic acid, which is extruded by the Monocarboxylic Acid Transporter (MCT). Deuterium Metabolic Imaging (DMI) is adept at monitoring the conversion of ^2^H-pyruvate to ^2^H-lactate (highlighted by the red arrow) during glycolysis, thereby offering insights into the metabolic flux of glycolysis in tumors (32). Similarly, DMI facilitates the evaluation of tumor cell death by tracking the metabolism of ^2^H-fumaric acid to ^2^H-malic acid (indicated by the blue arrow) during the TCA cycling (33, 34).

The critical advantage of DMI lies in its ability to utilize exogenous ^2^H labeling to capture multiple metabolic steps involved in glucose metabolism, as depicted in **Figure 2**. Furthermore, DMI facilitates the quantitative determination of biochemical kinetic constants, enhancing its utility in metabolic research. In contrast to ^1^H-MRSI, DMI does not necessitate the use of additional radio frequency (RF) pulses for the suppression of protonated water, due to the low abundance of natural deuterated water, leading to a low specific absorption rate (SAR) of RF power(14, 29). This will mitigate the rise in temperature during the patient examination process, thereby simplifying and enhancing the safety of DMI’s clinical application (35). Unlike PET, which faces challenges in brain tumor imaging (36), DMI has emerged as a valuable alternative (37). It overcomes the limitations posed by high glucose uptake in both normal brain tissue and malignant tumors, enabling precise imaging of malignancies (38). Although the combination of PET and CT provides a higher spatial resolution advantage, DMI offers a simpler and more convenient imaging method without any radiation exposure, making it safer for longitudinal studies (39). As a complementary technique to hyperpolarized ^13^C MRSI, DMI allows for the long-term monitoring of metabolic processes without requiring significant modifications to existing clinical magnetic resonance instruments. Additionally, the naturally low abundance of ^2^H (40) eliminates the need to suppress ^2^H signals from water and fat during MRSI (30).

## 3 Application of DMI in tumor metabolism

In this review, we highlight the potential of DMI in studying tumor metabolism, with a specific focus on glucose metabolism pathways. To provide a comprehensive perspective, **Table 1** provides an overview of various studies that have explored the application of DMI in the realms of metabolic imaging and treatment evaluation.

**Table 1:** A summary of the research on deuterium metabolic spectroscopy and imaging.

research	deuterium probe	species	field strength/T	Tumor type	Research content and conclusion
Glucose-lactic acid metabolism axis					
De Feyter et al. (29)	^2^H-glucose	rat/human	11.7T(rat)/4.7T(human)	Glioma	They successfully revealed a significant metabolic difference between the normal brain and tumor tissue through DMI.
Kreis et al. (32)	^2^H-glucose	mouse	9.4T	lymphadenoma	They proposed a dynamic DMI strategy toward a quantitative measurement of glycolytic metabolic flux in a lymphoma mice model using chemical kinetic analysis.
Tan et al. (41)	^2^H-glucose	acute myeloid leukemia (AML) cells	7T	Acute myeloid leukemia	They explored that DMI can be served as a non-invasive monitoring tool for early tumor treatment effects.
Markovic et al. (39)	^2^H-glucose	mouse	15.2T	pancreatic ductal adenocarcinoma (PDAC)	They proposed a kinetic model to describe the exchanges between kidney and tumor glucose, water, and lactate.
Veltien et al. (42)	^2^H-choline, ^2^H-glucose	mouse	11.7T	renal carcinoma	They revealed that DMI can simultaneously obtain two main tumor metabolic information (Glucose uptake and glycolytic metabolism and choline uptake).
SimõCheck that all equations and special characters are displayed correctly. es et al. (43)	^2^H-glucose	mouse	9.4T	Glioblastoma multiforme (GBM)	They developed a dynamic glucose-enhanced deuterium spectroscopy (DGE2H-MRS).
Fumaric-malic acid metabolism axis					
Hesse et al. (33)	^2^H-fumarate	mouse	7T	murine lymphoma (EL4)	They demonstrate that the ^2^Hmalate signal generated by ^2^H-fumarate monitored through DMI can be used to detect tumor cell death.
Hesse et al. (34)	^2^H-fumarate	mouse	7T	murine lymphoma (EL4)	They showed that cell death can be detected with similar sensitivity following oral administration of the ^2^H-labeled fumarate.
tumor telomere-related fields					
Batsios et al. (44)	^2^H-pyruvate	mouse	14.1T	glioblastoma	They found that TERT expression could be visualized *in vivo* using DMI.
Taglang et al. (45)	^2^H-glucose	mouse	14.1T	Astrocytoma	They found that DMI can be used as a new imaging method for the alternative lengthening of telomeres (ALT) pathway.
Others					
Hartmann et al. (46)	deuterated 3-O-Methylglucose (OMG)	rat	7T	breast cancer	They investigated the feasibility of using deuterated 3-O-methylglucose (OMG) to perform DMI on tumor-bearing mice in 2021 as a complementary to PET.

### 3.1 Tumor glycolysis metabolism

Metabolic reprogramming is a hallmark of almost every tumor cell, leading to a preference for utilizing glucose for glycolysis, even in the presence of oxygen (47, 48). The advent of DMI technology now enables the visualization and quantitative analysis of this metabolic reprogramming in tumors, as shown in **Figure 2**.

#### 3.1.1 Glucose-lactic acid metabolism axis

Several studies have demonstrated the potential of DMI in investigating the glucose-lactate metabolism pathway in tumors. De Feyter et al. successfully identified metabolic differences between normal brain tissue and tumor tissue using DMI in a rat glioma model, showing a higher level of the lactate/glutamine ratio in tumors (29). They also observed similar metabolic patterns and image contrast in two patients with a high-grade brain tumor after oral intake of ^2^H-labeled glucose. L. Tan et al. utilized DMI to observe metabolic changes in acute myeloid leukemia cells following cisplatin treatment, revealing higher lactate levels in AML cells compared to normal cells (41). The both studies jointly highlight the potential of DMI in tumor diagnosis from both *in vivo* and *in vitro* perspectives. Kreis et al. implemented a dynamic DMI approach to quantify glycolytic metabolic flux in a lymphoma mice model, revealing heterogeneity within tumors (32). Markovic et al. analyzed glucose metabolism in an orthotopic pancreatic cancer mouse model, unveiling differences in lactate clearance kinetics between two distinct pancreatic cancer subtypess (39). Simões et al. developed a dynamic glucoseenhanced deuterium spectroscopy (DGE2H-MRS) to evaluate the glucose metabolic turnover rate in mice with two different pathological types (GL261 and CT2A cells) of glioma by differential glycolysis and mitochondrial oxidation(43). n addition to providing insights into glycolysis metabolism, DMI also offers crucial information regarding another key aspect of tumor metabolism: choline uptake. Veltien et al. utilized DMI to monitor and image ^2^H-choline and ^2^H-glucose signals in subcutaneous kidney tumors in mice (42).

Tumor cells majorly obtain energy from anaerobic glycolysis resulting in lactate accumulation within the tumor tissue (12, 49). Elevated lactate levels in tumors are associated with cancer metastasis, progression, recurrence, and poor prognosis (49). Tumor cells not only use lactate as an energy source, but also shuttle it to adjacent cancer cells, stroma, and vascular endothelial cells, inducing metabolic reprogramming (50). Notably, glycolytic enzymes such as hexokinase2 (HK2)2 and pyruvate kinase (PK)M2 have been extensively studied for their correlation with cancer cell motility and invasive capacity (51–55). The integration of macroscopic imaging of tumor metabolism using DMI, in combination with Western blot analysis and other techniques, holds the potential to explore metabolic markers related to tumor metastasis and provide valuable assistance in tumor staging and prognosis.

#### 3.1.2 Fumaric-malic acid metabolism axis

In the tricarboxylic acid cycle of aerobic metabolism, fumarate is converted to malate catalyzed by the enzyme fumarase (or fumarate hydratase, EC 4.2.1.2) (56). Monitoring the malate signal generated from fumarate metabolism can provide insights into the early response of tumors to treatment and other pathological conditions associated with cell death, such as toxic injury or ischemia (57–61). While hyperpolarized ^13^C MRSI has demonstrated an increase in malate signal in treated tumor tissues due to tumor cell necrosis, researchers are exploring the potential of DMI as a cost-effective and more straightforward imaging tool for monitoring the fumarate-malate metabolism process in tumors (**Figure 2**) (56).

Hesse et al. (33) utilized DMI in a 7-Tesla MR scanner to monitor the metabolized ^2^H-malate signal in lymphoma cells and tumor-bearing mice before and after treatment with etoposide. Their findings suggest that DMI could serve as a noninvasive imaging tool to monitor early tumor response to therapy. Building on this research, they used DMI to track the malate signal in tumor tissue before and after treating tumor-bearing mice with etoposide and compared the effects of oral administration and intravenous administration of deuterated fumaric acid (34). This study discovered that ^2^H-fumarate, a cell death marker, could be detected with similar sensitivity through different administration methods. These findings provide a promising prospect for the clinical use of DMI in monitoring the early response of tumor patients to oral anti-tumor drugs.

These studies underscore the potential of DMI as a valuable tool for detecting tumor cell death by monitoring the conversion of fumarate to malate. DMI offers advantages over ^13^C hyperpolarized MRSI in terms of cost-effectiveness and ease of implementation. Consequently, DMI shows promise as a non-invasive imaging technique for monitoring cell death and early response to tumor treatment.

### 3.2 Application of DMI in tumor telomere-related fields

**Figure 3:**
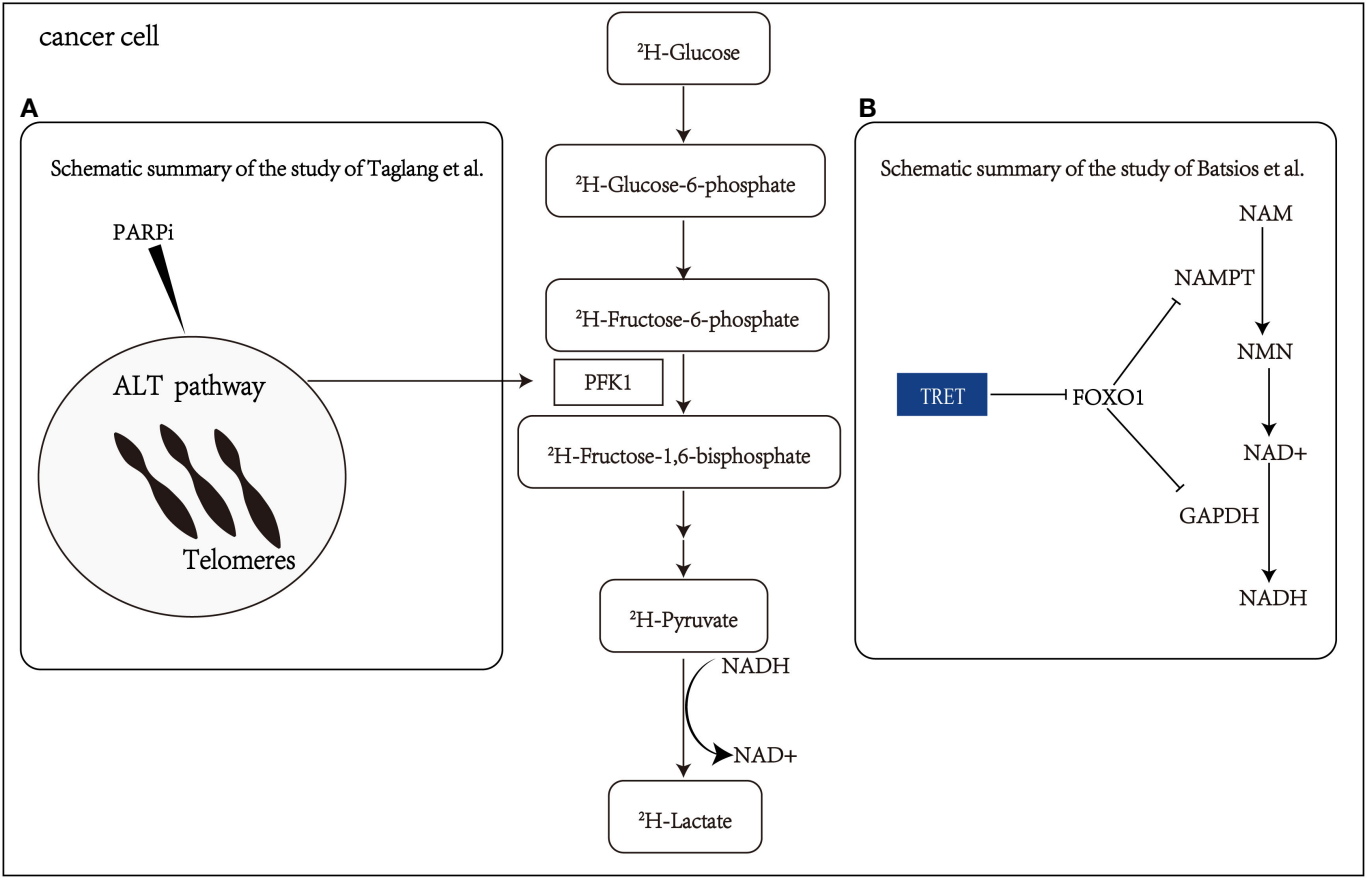
Schematic summary of the study of Batsios et al. Batsios et al. (44) and Taglang et al. Taglang et al. (45) **(A)** The alternative lengthening telomeres (ALT) pathway elevates glycolytic flux via enzyme phosphofructokinase-1(PFK1). This metabolic reprogramming and response to PARPi (polymerase inhibitors) that cause telomeric fusion in ALT cells can be non-invasively monitored via quantification of lactate production from 2H-glucose. **(B)** Schematic representation elucidating the intricate relationship among telomerase reverse transcriptase (TERT), FOXO1 (a member of the Fox family of transcription factors), nicotinamide phosphoribosyl transferase (NAMPT), glyceraldehyde-3-phosphate dehydrogenase (GAPDH), nicotinamide (NAM), nicotinamide mononucleotide (NMN), nicotinamide adenine dinucleotide (NADH), and pyruvate flux to lactate in TERT-positive (TERT+) cancer cells.

In addition to the Warburg effect, continuous cell division is a hallmark of advanced malignancy, often linked with the reactivation of telomere reverse transcriptase (TERT) (62). A recent study by Batsios et al. (44) has demonstrated that DMI can evaluate TERT expression *in vivo*, revealing a TERT-FOXO1 axis in cancer metabolic reprogramming. This connection sheds light on the role of TERT in the glycolytic pathway, particularly in the conversion of pyruvate to lactate (**Figure 3B**). The noninvasive detection of metabolic changes related to TERT expression through DMI holds significant promise. Further studies should aim to validate these findings in clinical settings and explore the clinical utility of DMI, providing new insights into the intricate relationship between TERT expression and cancer metabolism.

Diagnosing and evaluating the treatment response of astrocytoma can be challenging, especially in distinguishing tumor progression from pseudoprogression (63). Taglang et al. (45) have demonstrated that DMI could serve as a novel imaging technique for studying the alternative lengthening telomeres (ALT) pathway in astrocytoma. This telomerase-independent telomere maintenance mechanism involves metabolic reprogramming that increases glycolytic flux in astrocytoma (**Figure 3A**). The study utilized DMI to visualize glycolysis flux in astrocytoma mouse models, confirming the relationship between the ALT pathway and glycolytic flux through genetic and pharmacological approaches. These findings highlight the value of DMI in visualizing ALT-related metabolic reprogramming in astrocytoma and its potential for diagnosing and evaluating treatment response. Additionally, DMI exhibits the ability to detect early changes in tumor lactate signal, making it effective in identifying pseudo-progression.

These studies provided valuable insights into the telomere-related aspects of tumors and proposed a promising approach for metabolic imaging in cancer patients applicable in clinical practice. A biological link between tumor telomeres and glycolytic metabolism is established through the TERT or ALT pathway. Gene set enrichment analysis has further revealed enhanced glycolysis in adrenocortical carcinoma with high TERT levels (64). Overall, telomere maintenance is a hallmark of cancer and is often achieved through the reactivation of TERT or ALT pathways, which are associated with the glycolysis (45, 64–66). DMI offers a non-invasive and clinically translatable biomarker for evaluating the telomere-related expression in tumors, providing indirect but valuable imaging insights into the TERT and ALT pathways.

### 3.3 Other applications: new tracer in DMI-OMG

Deuterated 3-O-methylglucose (OMG) has emerged as a promising tracer in DMI, offering an alternative to PET for quantifying glucose uptake (46). Unlike ^18^FDG used in PET, OMG is a non-metabolizable glucose analog that is transported similarly to D-glucose. Hartmann et al. (46) conducted a study to explore the potential of OMG-DMI as a non-ionizing radiation alternative for tumor imaging. Their results demonstrated the feasibility of using OMG-DMI as a viable method for specific detection and quantification of glucose uptake in tumors.

## 4 Future development in DMI for tumor immunotherapy

Recent advancements in tumor immunotherapy have yielded significant clinical breakthroughs for numerous tumor patients (67–69). Infiltrating immune cells (e.g., T cells, B cells, neutrophils, etc.) undergo metabolic changes that can impact tumor progression and antitumor function. These metabolic alterations often involve increased glycolysis and lactate accumulation (70). However, the tumor microenvironment poses challenges to effective anti-tumor immunity due to hypoxia, acidity, and nutrient depletion (71). Studies have highlighted that highly expressed glycolytic genes can hinder the effectiveness of immunotherapy, while targeted glycolytic drugs (e.g., dimethyl fumarate, 2-deoxyglucose, diclofenac, etc.) have been found to enhance immunotherapy efficacy by promoting IFN-*γ* production in immune cells (72–74). Strategies aimed at inhibiting tumor cell glycolysis are actively under investigation to improve immunosurveillance and control tumor growth. Real-time visualization of tumor glycolysis can play a crucial role in treatment planning and monitoring the response to immunotherapy, thus enhancing its safety and effectiveness. DMI has been demonstrated as a simple and efficient imaging tool capable of real-time monitoring ^2^H-glucose and ^2^H-lactate signals to visualize the tumor glycolytic metabolism De Feyter et al. (29); Tan et al. (41). Consequently, DMI is expected to emerge as a non-invasive and promising imaging tool in tumor immunotherapy and targeted therapy.

## 5 Discussion

This review provides an overview of DMI’s applications in various tumor metabolic processes. The primary focus of most studies has been on the metabolic reprogramming of tumors, especially the Warburg effect. These investigations have successfully harnessed DMI for non-invasive imaging of tumor glycolysis metabolism. DMI has also shown potential in detecting the malate signal produced by fumarate metabolism within tumor tissue, enabling the non-invasive evaluation of early tumor response to treatment and cell death. Furthermore, the reactivation of the TERT andor ALT pathways, critical for maintaining tumor telomeres, has been biologically linked to glycolysis. Researchers have utilized DMI for TERT and ALT pathway imaging, allowing non-invasive evaluation of telomere-related expression in tumors. As a result, DMI holds the potential to become a convenient and efficient imaging tool in clinical practice, providing non-invasive evaluation of tumor metabolism.

Recent studies have demonstrated that T cell effector functions and augments immunotherapy were correlated with restricted glycolysis (72–74). This makes DMI a valuable tool for evaluating treatment response after immunotherapy, as it can image and monitor tumor glycolytic metabolism. DMI can aid in pinpointing optimal time windows for immunotherapy and improving the precision of clinical interventions. Additionally, lactate accumulation in tumors is associated with cancer metastasis and prognosis (49, 75). DMI enables non-invasive visualization of the entire metabolic process within tumors, providing valuable insights into tumor prognosis and staging. Recent research has also unveiled a mechanistic connection between tumor glycolysis metabolism and telomere elongation, which can be evaluated using DMI (44, 45). As a macroscopic imaging tool, DMI complements and validates microscopic perspectives, such as proteins, DNA, RNA, etc., offering valuable information on metabolic changes.

Furthermore, DMI can provide complementary perspectives and evidence when used in conjunction with other techniques, such as ^13^C MRSI (57–61). Labeled malate production in ^13^C MRSI has demonstrated the *in vivo* imaging of cell death (57–61). As our understanding of tumor and immune cell metabolism advances in cancer progression and immunotherapy, DMI exhibits great potential in cancer diagnosis and treatment evaluation (76), DMI exhibited the great application prospects in cancer diagnosis and treatment evaluation. While DMI is rapidly advancing in cancer research, there are still several challenges to be addressed for its clinical translation. These challenges encompass the development of reliable methods for quantifying deuterium substance injection, ensuring the safety of DMI probes, and advancing the hardware development of clinical systems. A standardized and comprehensive set of indicators and protocols for DMI can be adopted from relevant diagnostic and evaluation approaches of PET. This standardization would be crucial for advancing further clinical research in cancer using DMI.

## 6 Conclusion

In conclusion, DMI emerges as a powerful and promising tool for investigating tumor metabolism. Its unique capabilities allow non-invasive imaging of various metabolic pathways within tumors, encompassing glycolysis, fumarate metabolism, and telomere-related pathways. DMI can provide invaluable insights into tumor biology, treatment response, and prognosis assessment. Moreover, its potential application in immunotherapy holds the promise of evaluating early treatment responses and optimizing therapeutic strategies. However, it is imperative to recognize that further research is essential to address challenges related to quantification, safety, and hardware development. With ongoing advancements, DMI stands poised to evolve into a convenient and efficient imaging tool in clinical practice. Its contributions will play a pivotal role in the advancement of precision medicine and the improvement of patient outcomes across the spectrum of cancer diagnosis and treatment evaluation.

## Author contributions

JW: Conceptualization, Investigation, Writing – original draft, Writing – review & editing, Methodology. YG: Conceptualization, Writing – review & editing. HC: Conceptualization, Writing – review & editing. PS: Conceptualization, Methodology, Supervision, Visualization, Writing – review & editing. XZ: Conceptualization, Formal Analysis, Supervision, Visualization, Writing – review & editing. TY: Project administration, Resources, Supervision, Writing – review & editing. LL: Investigation, Visualization, Writing – review & editing. FP: Visualization, Writing – review & editing. LY: Visualization, Writing – review & editing.
